# Thiol dependent intramolecular locking of Orai1 channels

**DOI:** 10.1038/srep33347

**Published:** 2016-09-14

**Authors:** Dalia Alansary, Barbara Schmidt, Kathrin Dörr, Ivan Bogeski, Heiko Rieger, Achim Kless, Barbara A. Niemeyer

**Affiliations:** 1 Molecular Biophysics, Saarland University, 66421 Homburg, Germany; 2 Department of Biophysics, Saarland University, 66421 Homburg, Germany; 3 Department of Theoretical Physics, Saarland University, 66041 Saarbrücken, Germany; 4 Gruenenthal Innovation, Drug Discovery Technologies, Gruenenthal GmbH, 52078 Aachen, Germany

## Abstract

Store-operated Ca^2+^ entry mediated by STIM1-gated Orai1 channels is essential to activate immune cells and its inhibition or gain-of-function can lead to immune dysfunction and other pathologies. Reactive oxygen species interacting with cysteine residues can alter protein function. Pretreatment of the Ca^2+^ selective Orai1 with the oxidant H_2_O_2_ reduces I_CRAC_ with C195, distant to the pore, being its major redox sensor. However, the mechanism of inhibition remained elusive. Here we combine experimental and theoretical approaches and show that oxidation of Orai1 leads to reduced subunit interaction, slows diffusion and that either oxidized C195 or its oxidomimetic mutation C195D located at the exit of transmembrane helix 3 virtually eliminates channel activation by intramolecular interaction with S239 of transmembrane helix 4, thereby locking the channel in a closed conformation. Our results demonstrate a novel mechanistic model for ROS-mediated inhibition of Orai1 and identify a candidate residue for pharmaceutical intervention.

Reactive oxygen species (ROS) are typically defined as biologically reactive molecules or ions formed by reduction of oxygen. Sequential reduction of oxygen leads to the formation of a number of ROS including superoxide, hydrogen peroxide, hydroxyl radical and hydroxyl ion. Extracellular stimuli e.g. growth factors^1^, cytokines^2^ and pathogens^3^ as well as endogenous stimuli e.g. hypoxia^4^ can induce generation of ROS mainly via activation of NADPH oxidases (NOX) and as a byproduct of active mitochondrial respiration. Extracellular ROS can be taken up by cells through aquaporins^5^ and are degraded in the cytosol through the action of enzymes such as superoxide dismutase, catalase or the glutathione reductase system^6^ or in the extracellular space through membrane associated catalases and superoxide dismutases (e.g. SOD3^7^). While low concentrations (likely in the nanomolar to low micromolar range) of ROS trigger or influence local signaling cascades, alter gene expression and combat bacterial infections utilizing specialized enzymes (NOX), higher concentrations can also cause damage to nucleic acids, proteins or lipids (see reviews^8^,^9^). Major targets of ROS-induced modification of proteins are reactive cysteine residues. A reactive cysteine contains a thiolate group (S-) which reacts with H_2_O_2_ with rates ranging from 10 to 10^5^ M^−1^s^−1^, depending on their local environment, while the thiol groups (SH) do not react physiologically with H_2_O_2_ unless the reaction is catalyzed^10^. The thiolate undergoes reversible (to sulfenic) or irreversible (to sulfinic and sulfonic acid) covalent modifications upon oxidation. Additionally, mild oxidation can induce reversible cysteine disulfide bond formation and thus prevent further irreversible cysteine modifications^11^. Oxidation and consequent structural changes such as intermolecular cross linking can modify the function of the target proteins^9^,^12^. Research in the last two decades provided evidence that ROS represent an important and physiologically relevant direct or indirect regulators of several ion channels: while oxidation results in activation of TRPM2^13^, TRPV1^14^,^15^, TRPV4^16^ and TRPA1^17^, prevents inactivation of Nav channels^18^, ROS inhibit members of Kv^19^,^20^, Cav^21^ and CRAC^22^,^23^ channel families.

Orai1 proteins form the major ion conducting units mediating the Ca^2+^ release activated Ca^2+^ current (I_CRAC_) in immune cells among many other cell types. These currents are activated by interaction with ER-resident Ca^2+^ sensor molecules STIM that translocate to plasma membrane-near regions in response to store depletion, inducing to store operated Ca^2+^ entry (SOCE). We have previously shown that preincubation with ROS prevent activation of Orai1, but are unable to inhibit the channel complex once it is activated^22^ in contrast to other I_CRAC_ blockers^24^,^25^. The inhibition is mainly mediated through the reactive cysteine C195 at the exit of transmembrane region 3 (TM3) of Orai1, a residue that is not conserved in the paralogue Orai3, which currents are not inhibited by oxidation^22^. Electrophilic addition to Orai1’s C195 is also the main reason for the inhibitory effect of curcumin and caffeic acid phenethyl ester (CAPE) on I_CRAC_^26^. Differentiation of naïve CD4 T helper cells into effector cells upon TCR stimulation is accompanied by both upregulation of the ROS resistant paralogue Orai3 and of intracellular antioxidant enzymes. Concomitantly, cytokine production and proliferation of effector cells become more resistant to inhibition by H_2_O_2_ and the inhibition of SOCE shows an increased IC_50_ when compared to naïve cells^22^. Differential ROS resistance of SOCE due to altered Orai3 expression has also been confirmed for primary prostate epithelial cells versus cells derived from prostate cancers^27^ and for ROS producing monocytes, where upon bacterial challenge, the Orai3/Orai1 ratio shifts and allows for a feedback adaptation optimizing Ca^2+^ dependent ROS production^23^. Although the stoichiometry of Orai1/Orai3 heteromeric channel proteins *in vivo* is not known and Orai3 mRNA is usually less abundant, the addition of a single subunit of Orai3 to a concatenated heteromer is sufficient to eliminate ROS sensitivity^28^.

What remains unclear is the molecular mechanism by which oxidation of C195 at the exit of the third transmembrane helix (TM3) of Orai1 prevents its activation and our current work aims at unravelling this mechanisms by combining experimental and theoretical approaches.

## Results and Discussion

### Inhibition of Orai1 mediated I_CRAC_ is irreversible and less accessible to MTSES

As we and others have shown, application of H_2_O_2_ does not inhibit already activated I_CRAC_^22^,^29^ and inhibition requires preincubation before activation^22^. To gain insight into the nature of the chemical modification resulting from pretreatment with H_2_O_2_, we tested the reversibility of the H_2_O_2_ effect in stable STIM1 expressing HEK293 (HEKS1) cells overexpressing Orai1. The current measured in cells pretreated with H_2_O_2_ showed a 54% inhibition of I_CRAC_ compared to control cells, in compliance with our previous results. Neither short (<30 min) nor prolonged (>30 min) treatment of the cells with an equimolar concentration of a reducing agent (Dithiothreitol, DTT) after the initial oxidizing stimulus was able to reverse the inhibition (Fig. S1a,b) implying that H_2_O_2_ pretreatment leads to an irreversible oxidation into a sulfinic or sulfonic and not into the reversible sulfenic acid derivative of the sensor cysteine thiol group. As the DTT treatment was also applied prior to store depletion, this irreversible oxidation takes place prior to the interaction with STIM1 molecules. An I_CRAC_ inhibition is also observed by other oxidizing agents whereby the inhibition seen by the membrane permeant and smaller alkylthiosulfonate MTSEA is bigger than that seen with the membrane impermeant larger MTSES indicating that the ROS sensors show limited accessibility from the extracellular side (Fig. S1c,d).

### Inhibition is not due to a reduced STIM1-Orai1 interaction

**Figure 1:**
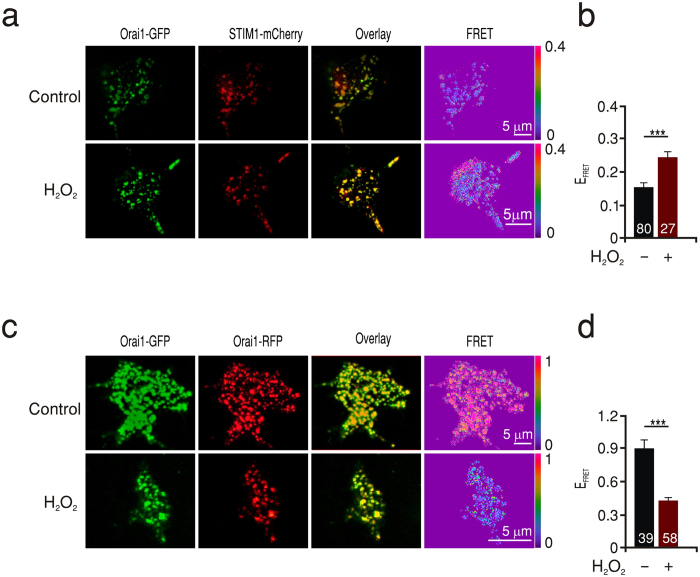
ROS effects on STIM1-Orai1 and Orai1-Orai1 interactions. (**a**) Images showing HEKWT cells expressing Orai1-GFP (green) and STIM1-mCherry (red), the corresponding overlay and FRET images (LUT 0–0.4) obtained from control (upper panel) or H_2_O_2_ pretreated (lower panel) cells after Tg induced store depletion. (**b**) Quantification of FRET efficiency (EFRET) measured in a. (**c**) Images showing HEKS1 cells expressing Orai1-GFP (green) and Orai1- RFP (red) and the corresponding overlay and FRET images (LUT 0–1) form cells treated like in a. (**d**) Quantification of FRET efficiency (EFRET) measured from cells in c.

Because initiation and development of I_CRAC_ requires the interaction of Orai1 with clustered STIM1 molecules at ER-PM junctions which leads to their subsequent trapping and activation, we first controlled whether oxidants affect the ability of Orai1 to interact with STIM1. We used a combined approach utilizing Total Internal Reflection Microscopy (TIRFM) to observe signals within 150 nm of the plasma membrane and Förster Resonance Energy Transfer (FRET) microscopy to measure the interaction between Orai1-GFP and STIM1-mCherry after store depletion. Interestingly, pre-incubation of cells expressing both proteins with H_2_O_2_ significantly promoted Orai1-STIM1 FRET efficiency (E_FRET_) values to 0.24 ± 0.03 compared to 0.15 ± 0.01 in control conditions (Fig. 1a,b). Our earlier study showed that an Orai1 triple cysteine mutant (C126S; C143S; C195S: TM) is not inhibited by preincubation with H_2_O_2_, ruling out a significant effect of externally added H_2_O_2_ on the principal ability of STIM1 to activate Orai1^22^. Together, these results indicate that the inhibition we observe with preincubation is likely independent of the redox state of STIM1.

We used the same TIRF-FRET approach to measure the effect of oxidation on the interaction between subunits of Orai1 in cells co-expressing Orai1-GFP and Orai1-RFP. Using C-terminally tagged constructs we observed Orai1-Orai1 E_FRET_ values of 0.89 ± 0.08 in control conditions (Fig. 1c,d). These values are higher than values previously published, obtained with N-terminally tagged constructs and not measured within the TIRF plane^30^,^31^,^32^. In contrast to the interaction with STIM1, Orai1-Orai1 subunit interaction was reduced by H_2_O_2_ pretreatment to 46% of the control value (Fig. 1). The reduced Orai subunit interaction may explain the increased STIM1-Orai1 interaction as a reduced interaction between two adjacent Orai1 C-termini could facilitate STIM1-Orai1 FRET^33^. However, we cannot deduce the subunit stoichiometry from FRET experiments.

To investigate if the reduced subunit interaction has an influence on the apparent open probability of the channel and to gain insight into the Ca^2+^ dependent inactivation which is also dependent on the relative ratio of STIM1:Orai1^34^, we measured the instantaneous tail currents at −100 mV in response to voltage steps in the range of −160 to + 80 mV. When plotted against voltage, the normalized apparent open probability shows that both control and pre-incubated cells show inactivation (apparent P_o_ below 1) at negative voltages with no significant differences between treated and untreated cells (Fig. S2a). This indicates that oxidation did not significantly affect fast calcium dependent inactivation (FCDI) or dramatically shifted the STIM1:Orai1 ratio. The finding that oxidation also inhibited divalent free currents (−265.3 ± 60.4 pA/pF control, n = 6 vs −91.5 ± 60.2 pA/pF, treated n = 6), implies that oxidation does not directly affect the properties of the selectivity filter or ion conduction pathway.

**Figure 2:**
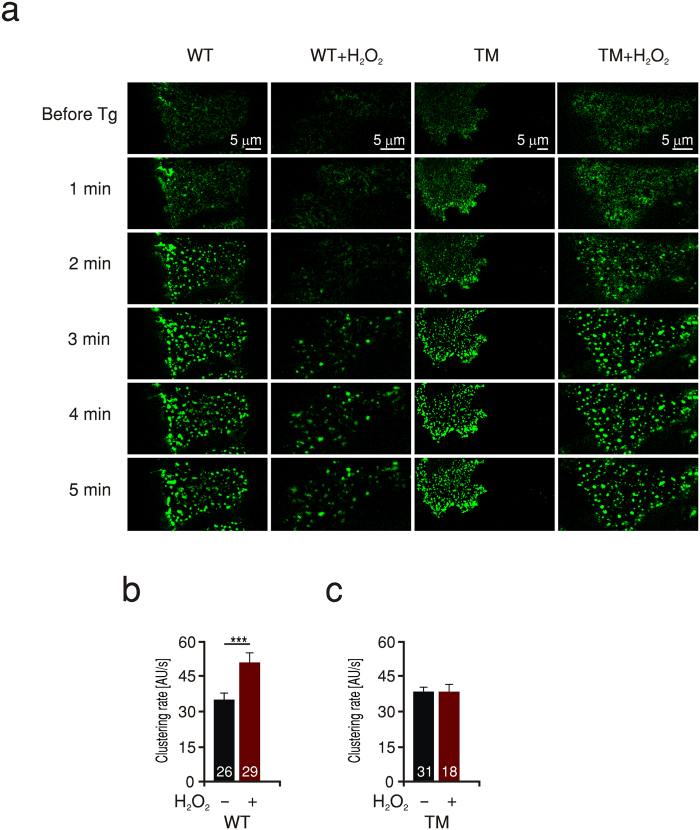
ROS slow down clustering of WT but not TM Orai1 channels. (**a**) Time-lapse TIRF images from cells expressing WT Orai1-GFP (WT) or triple cysteine mutant Orai1-GFP (TM) from control or H_2_O_2_ pretreated cells (WT/TM + H_2_O_2_). Base line images were obtained in the resting state (Before Tg) and the rate of cluster formation was monitored over 5 min at 1 Hz frequency. Rows depict representative images at a minute interval for each condition. (**b**) Average clustering rate from cells expressing WT Orai1-GFP with (red) and without H_2_O_2_ (black) pretreatment measured in a, first and second columns. (**c**) Average clustering rate from cells expressing TM Orai1-GFP with (red) and without H_2_O_2_ (black) pretreatment measured in a, third and fourth columns.

**Figure 3:**
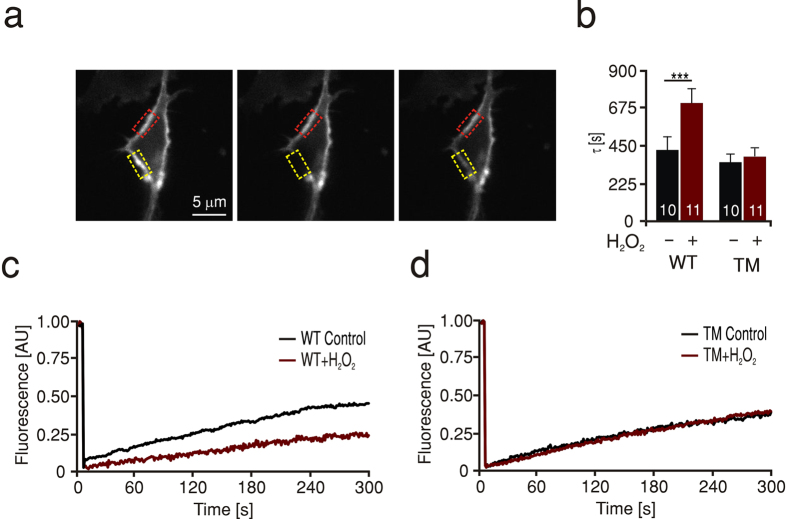
ROS slow down diffusion of WT but not TM Orai1 channels. (**a**) Images of a representative HEKS1 cell expressing Orai1-GFP before (left), immediately following (middle) and 5 min after recovery from a 7 s photobleaching laser exposure directed to the area lined with the dashed yellow line. The dashed red line defines a control non-bleached area used to correct for autobleaching (see methods). (**b**) Average rate constant (τ) of recovery after photobleaching analysed from of HEKS1 cells expressing WT or TM Orai1-GFP in control conditions (black) or after pretreatment with H_2_O_2_ (red). (**c**) Representative traces of HEKS1 cells measured in b and expressing WT Orai1-GFP. (**d**) Representative traces of HEKS1 cells measured in b and expressing TM Orai1-GFP.

Does a defective Orai1-Orai1 subunit interaction due to oxidation manifest itself also in a slower rate of Orai1 cluster formation? To address this, we applied TIRF microscopy to HEKS1 cells expressing Orai1-GFP and monitored the rate of Orai1-cluster formation during and after store depletion. The rate of Orai1 cluster formation in cells pretreated with H_2_O_2_ was significantly lower than that in control cells implying that oxidized channels form clusters slower than non-oxidized ones (Fig. 2a,b). To specifically correlate the slowed clustering rate with oxidation of Orai1, we examined the effect of H_2_O_2_ treatment in cells expressing Orai1 triple cysteine mutants (TM) which are insensitive to oxidation^22^. Indeed, the clustering rate of these mutant channels was not affected by pretreatment with H_2_O_2_ indicating that the decrease seen in the rate of clustering is mediated through cysteine oxidation of Orai1 (Fig. 2a,c). In these experiments we did not visualize STIM1, however we do not exclude the possibility of a clustering effect of H_2_O_2_ on STIM1 as documented by Hawkins and colleagues^35^. Nevertheless, the lack of H_2_O_2_ effects on the Orai1 triple cysteine mutant rules out involvement of STIM1 in the inhibitory effect. We then investigated whether the effect of H_2_O_2_ on the rate of Orai1 cluster formation seen in thapsigargin treated cells may be based on an altered diffusion of channel subunits also in the resting state. We therefore conducted “Fluorescence Recovery After Photobleaching” (FRAP) microscopy on cells expressing Orai1-GFP and monitored recovery of fluorescence after laser induced photo-bleaching in unstimulated cells with and without pretreatment with H_2_O_2_. Figure 3c shows the normalized fluorescence recovery as a function of time. The time course of fluorescence recovery for each cell was fitted by a single exponential and the rate constant tau (τ) was deduced and averaged in Fig. 3b. Pretreatment of cells expressing Orai1 (WT) with H_2_O_2_ resulted in an increase in τ to 710 ± 71 s compared to 427 ± 70 s in untreated cells, indicating a slowed diffusion of the oxidized proteins. In contrast, diffusion of the triple cysteine mutant channels (TM) was not significantly influenced by H_2_O_2_ pretreatment (Fig. 3b,c) again confirming the specificity of the effect of H_2_O_2_ on the presence of cysteines within Orai1 channels.

To assess whether the observed effects are sufficient to explain the dramatic oxidant inhibition of Orai1 mediated currents, we implemented these altered diffusion rates into a modified version of the stochastic reaction diffusion model that we have previously reported^36^,^37^. In contrast to the earlier version, our model now assumes that I_CRAC_ is mainly carried by STIM1 gated hexameric^38^ channels (highest open probability) with transitional tetrameric channels having a lower open probability and both being generated by trapping Orai1 dimers into multiple regions of clustered STIM1 (for schematic representation, see Fig. S2b). A more detailed description of the stochastic model can be found in the supplemental methods. Upon trapping, the linear combination of dimeric, tetrameric or hexameric channels yields the largest current at an Orai1:STIM1 ratio of 0.5 (Fig. S2c, black trace) and shows that the time course of current development closely matches the experimental data (Fig. S2d compare black trace with WT Control data). The model predicts that a decrease of only the diffusion constant by a factor of 0.66 does not change the size and kinetics of predicted current (Fig. S2d, green trace). However, combining the decreased diffusion rate constant of Orai1 with a 2.25 fold decrease in channel subunit interaction and a 1.4 fold increase of the STIM1-Orai1 interaction from the experimentally measured parameters upon H_2_O_2_ treatment (altered rates, Tables S1 and 2) results in a reduction of the currents (Fig. S2d, blue trace). The model thus predicts that reduced Orai1 diffusion rate constant together with a decreased subunit interaction and enhanced Orai1-STIM1 interaction may partially explain the reduced I_CRAC_ observed in the experiments following H_2_O_2_ oxidation of C195, but also gave us a strong incentive to search for additional experimental approaches to fully understand the phenotype.

### An oxidomimetic mutation phenocopies ROS – mediated current inhibition

**Figure 4:**
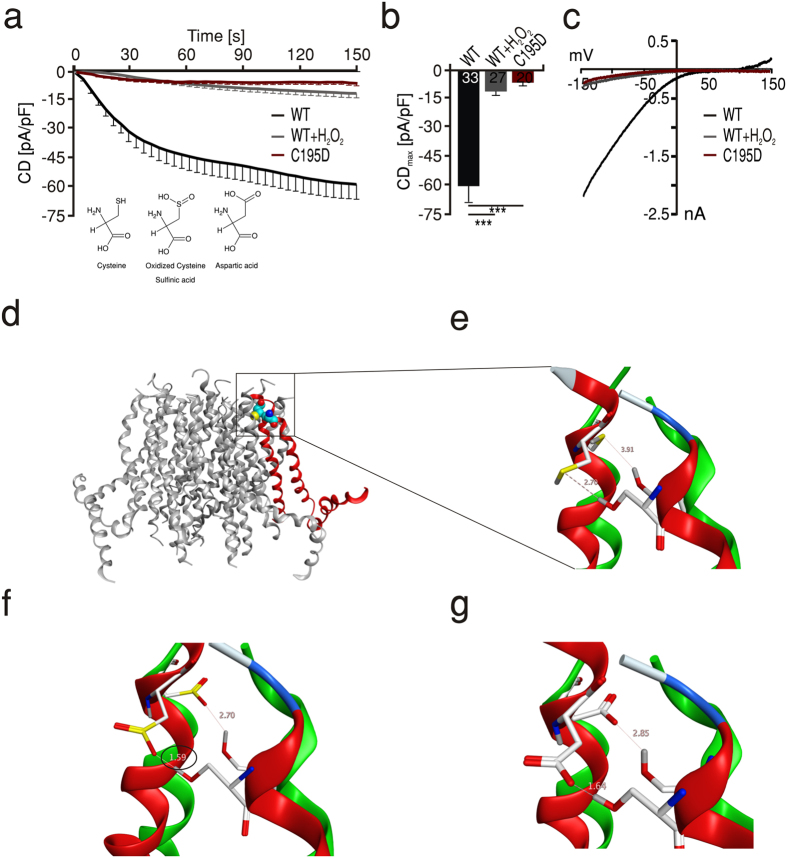
An oxidomimetic mutation phenocopies ROS-mediated inhibition. (**a**) Average traces showing whole cell current density (CD) over time extracted at −130 mV in HEKS1 cells expressing WT Orai1 (black) or C195D Orai1 (red) measured in control conditions and compared to currents measured from cells expressing WT Orai1 after pretreatment with 1 mM H_2_O_2_ (grey). (**b**) Average CD_max_ recorded from cells measured in a. (**c**) Current-voltage (I-V) relationship of representative cells recorded in a. (**d**) Structure homology model of hOrai1 with TM3 and TM4 highlighted in red. (**e**) Overlay of predicted structure of the enlarged zone in D in the closed (red) and open (green) states of the channel showing an increase from 2.7 to 3.91 A° of the predicted distance between sulfur atom of C195 and the hydrogen atom in S239 upon opening of a non-oxidized channel. (**f**) Oxidation of C195 to sulfinic or sulfonic acid derivative shortens the distance between C195 and S239 to 1.59 A° in the closed state and after transformation towards an open state to 2.7 A° (**g**) At position 195, replacement of cysteine with an aspartate residue results in displacement to S239 that mimic oxidation.

To obtain a better molecular understanding of the effects of an oxidized C195, we sought to unify the redox state of only Orai1 by generating a mutation that mimics the oxidation the thiol group of C195. In comparison to a reactive cysteine that has been irreversibly oxidized to sulfinic/sulfonic acid, an aspartate residue is the closest species regarding the structure and charge of the side group (see inset in Fig. 4a). We, therefore, mutated C195 of Orai1 to an aspartate (C195D), to introduce an oxidomimetic mutation. Indeed, the oxidomimetic Orai1 channels mediated an I_CRAC_ that closely mimicked the effect of H_2_O_2_, displaying current densities as small as those mediated by WT Orai1 in H_2_O_2_ pretreated cells (Fig. 4a–c). To further predict possible residues interacting with C195 we built a homology model of human Orai1 on the basis of the published structure of *Drosophila* Orai1^38^ as described in the materials and methods section. We also ran molecular dynamics (MD) simulation and included docking of the hSTIM1 SOAR/CAD domain into our simulation of the conformational change towards opening of the channel. Figure 4d shows a side view of the hOrai1 model with highlighted transmembrane helices TM3/TM4 (red) and the location of C195 and its nearest neighbor in TM4, S239. The relative distances between these residues are shown in the enlarged view (Fig. 4e) that has been generated by a structural overlay of hOrai1 in the closed state (red) on the basis of our homology model and towards the open state (green), derived from the molecular dynamics simulation. Upon activation, the interspace of C195 and the closest candidate residue of TM4, S239, revealed an increased distance from 2.70 to 3.91 Å between the hydrogen atom of S239 and the sulfur atom of C195 (Fig. 4e). The distance between C195 and S239 (2.70 Å) and electronegativity of the thiol group in C195 would not allow an interaction between these residues in the closed or in the open state (Fig. 4e). However, replacing the thiol group in the model by a sulfinyl or sulfonyl group, expected to be formed upon irreversible oxidation of C195, reduces the distance to S239 from 2.70 to 1.59 Å in the closed state. The shorter distance between C195 and S239 together with the lower pKa value of the sulfinic acid, which will lead to a deprotonated state under physiological conditions, enables formation of a stable hydrogen bond between the sulfinyl group and S239 in the closed state (Fig. 4f). However, if an equivalent transformation to the open conformational state (green) precedes oxidation, this does not allow for such an interaction due to the larger distance of 2.70 Å between C195 and S239. The structural comparison of the two conformational states of the channel may thus explain why H_2_O_2_ inhibits the closed but not the open state of Orai1.

**Figure 5:**
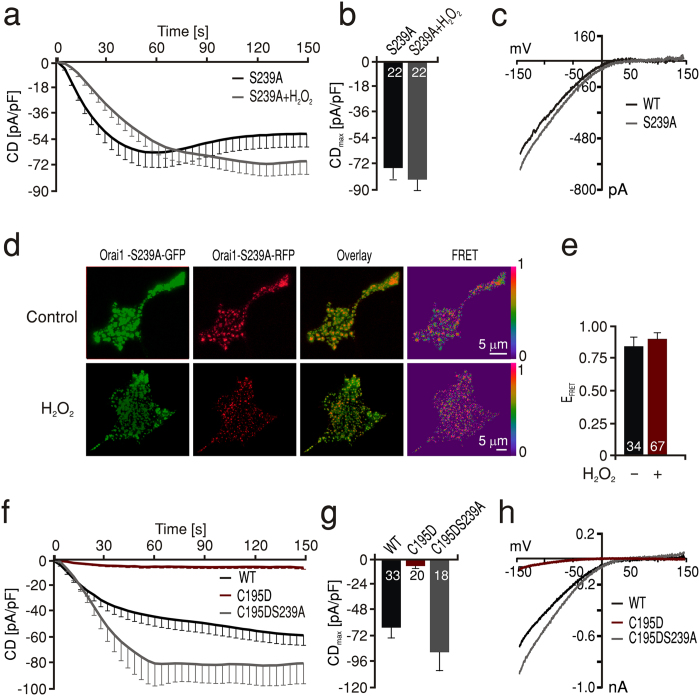
S239 is essential for ROS-mediated inhibition of current and Orai1 subunit interaction. (**a**) Average traces showing whole cell current density (CD) over time extracted at −130 mV in HEKS1 cells expressing S239A Orai1 measured in control conditions (black) or after pretreatment with 1 mM H_2_O_2_ (grey). (**b**) Average CD_max_ recorded from cells measured in a. (**c**) Current-voltage (I–V) relationship of representative cells recorded in a. (**d**) Images showing HEKS1 cells expressing Orai1S239A-GFP (green) and Orai1 S239-RFP (red), the corresponding overlay and FRET images (LUT 0–1) obtained from control (upper panel) or H_2_O_2_ pretreated (lower panel) cells after Tg induced store depletion. (**e**) Quantification of FRET efficiency (E_FRET_) measured in d. (**f**) Average traces showing whole cell current density (CD) over time extracted at −130 mV in HEKS1 cells expressing WT Orai1 (black), C195D Orai1 (red) or C195DS239A Orai1 (grey). (**g**) Average CD_max_ recorded from cells measured in f. (**h**) Current-voltage (I–V) relationship of representative cells recorded in f.

To test whether the observations derived from the molecular homology model of human Orai1 also account for the reduced current observed with the oxidomimetic mutant, we first mutated the position 195 into an Asp in the model. This mutation theoretically also allows the formation of a strong hydrogen bond between the two residues (1.64 Å) (Fig. 4g, red). Similar to WT, an equivalent transformation in the open conformational state does not allow such an interaction due to the larger distance of 2.85 Å (Fig. 4g, green). The model therefore implies that the small currents measured with the C195D mutant (Fig. 4a–c) are due to locking the channels in the inhibited state. If indeed S239 forms a stable hydrogen bond with oxidized cysteine 195 or with C195D and thereby locks the channel in a conformation that prevents movement of TM4 to be efficiently transmitted towards the ion conducting TM1, then S239 is necessary for the inhibition seen by H_2_O_2_ on WT Orai1. To test this hypothesis, we generated Orai1 S239A mutant channels and measured I_CRAC_ under control and oxidizing conditions. In line with our hypothesis, the S239A channels showed I_CRAC_ with characteristic I-Vs and large current densities that were not inhibited after pretreatment with H_2_O_2_ (Fig. 5a–c). Furthermore, we tested the redox sensitivity of the S239A subunit interaction using FRET. In agreement with the importance of this residue for the redox sensitivity of the Orai1, the S239A mutant showed no reduction of its FRET efficiency after H_2_O_2_ treatment with comparable FRET efficiency to the WT channel in control conditions (Fig. 5d,e). These results indicate that next to the redox sensor C195 of TM3, S239 of TM4 is also essential for ROS mediated inhibition of Orai1 and that the interaction between oxidized C195 and S239 can alter interacting protein interfaces to affect FRET. These findings also imply that the current inhibition seen with oxidomimetic mutant C195D is likely due to a stable hydrogen bond with S239 which, if prevented, should lead to a rescue of the small currents seen in C195D mutants. To test this hypothesis, we created double mutant C195D-S239A channels and measured I_CRAC_. Figure 5f–h shows that we observed a full recovery of I_CRAC_ in Orai1 C195D- S239A double mutants to a size that even slightly exceeded that of WT.

This data shows that the effect of intramolecular hydrogen bonding is likely the major mechanism by which H_2_O_2_ locks the channels in a configuration that prevents opening. To investigate the dependency of current density on the relative amount of pre-inhibited channel subunits, we included the inhibited channels as “locked” channels in our reaction diffusion model. Comparing different fractions of inhibited channels (Fig. S2c, O_inhibited_/O_total_), we clearly see a strong decrease in the steady state value of I_CRAC_ (at 600 s) for ratios of total Orai1 to STIM1 between 0 and 2 (Fig. S2c) that correlates with the increased fraction of O_inhibited_. Assuming a fraction of 0.95 inhibited channels the simulation now shows a strong reduction of current that resembles experimental data (Fig. S2d, red trace and symbols). The model also allows us to predict different degrees of inhibition depending on the fraction of inhibited channels.

In summary, the effects seen on the kinetics of cluster formation and diffusion are dependent on the presence of the cysteines of Orai1 and do not exclude an effect of oxidation of C126 or C143, but by themselves are not sufficient to explain the strong overall inhibition. Whereas the theoretical and experimental molecular analysis of the environment of C195 revealed that inhibition of Orai1 currents is due to the formation of intramolecular interactions between oxidized C195 in TM3 and S239 in TM4 that lock the channel in an inhibited conformation. Our findings are in line with the model proposed by Zheng and colleagues where Orai1 stepwise gating is mediated by initial interaction of STIM1 to the C-terminus of Orai1 that docks STIM1 to the channel and allows opening^39^, see also recent reviews by^40^,^41^. Our data shows that oxidation does not prevent docking of STIM1, however, docking is unable to induce sufficient movements of the transmembrane helices of Orai1 to allow widening of the pore lining TM1 region and thereby allowing ion conduction. In accordance with^42^, this demonstrates the critical importance of movement within TM3 and TM4 for gating of Orai1.

## Materials and Methods

### Cell Culture, Constructs and Transfection

Human embryonic kidney (HEK293, ATCC CRL-1573) WT or stably expressing STIM1 (HEKS1) cell lines were maintained in a 37 °C, 5%CO_2_ humidified incubator in corresponding growth medium. All *hOrai* constructs were subcloned into pCAGGS-IRES-GFP or directly GFP or RFP-tagged at the C-terminus in pMAX. To avoid affecting interaction of STIM1 with the plasma membrane by masking the C-terminal KK domain with fluorescent tags, mCherry was inserted at L599. All constructs were confirmed by sequencing. For transfection, the indicated amount of plasmid DNA was electroporated into HEK293 or HEKS1 cells with Nucleofector II according to the manufacturer’s instructions 24 h before measurements.

### Electrophysiology

Recordings were performed at room temperature in the tight-seal whole cell configuration. Linear voltage ramps from −150 mV to +150 mV were applied as in^36^. The pipette solution contained the following (in mM): 120 Cesium-glutamate, 3 MgCl_2_, 20 Cesium-BAPTA, 10 Hepes and 0,05 IP_3_ (pH 7,2 with CsOH). The bath solution contained (in mM): 120 NaCl, 10 TEA-Cl, 10 CaCl_2_, 2 MgCl_2_, 10 Hepes and Glucose (pH 7,2 with NaOH). Divalent free (DVF) bath solution contained the following (in mM): 120 NaCl, 10 TEA-Cl, 10 Hepes, 10 EDTA and glucose (pH 7.2).

### Apparent Open Probability Analysis

Normalized instantaneous tail currents in response to voltage steps to −100 mV after test pulses ranging from −160 to +80 mV were used to produce the apparent open probabilities (Po) according to^34^,^43^. To determine the amplitude of the instantaneous tail currents and to minimise the error due to cell-capacitance, tail currents were fitted with a single exponential function to the beginning of the −100 mV pulse.

### Fluorescence Recovery After PhNotobleach (FRAP)

Confocal imaging was performed on multibeam confocal scanner systems (VTinfinity-3, VisiTech Int., Sunderland, UK) using a 60× oil immersion objective. Images were acquired at room temperature in a 2 mM Ca^2+^-containing Ringer solution. After base line recording, HEKS1 cells transfected with 1 μg Orai1-GFP were exposed to a 491 nm laser with 100% intensity directed to a predefined region within the cell for 7 s to induce photobleaching. Initial fluorescence and recovery after photobleach were measured by scanning across the bleached region at a laser intensity of 40% for 100 ms and a frequency of 1 Hz. Recovery was monitored over 5 min. After acquisition, data was transferred into ImageJ software (W. Rasband, NIH, USA) for further processing. To correct for autobleaching effects, fluorescence intensity was integrated over a region overlaying the photobleached profile and a non-bleached control region. The change in ratio of the two regions was plotted over time. The relative intensity before and immediately after photobleaching were set to 1 and 0, respectively. Time constants (τ) were calculated for each cell by fitting the resulting curve with an exponential function using Igor software (Wavemetrics).

### Förster Resonance Energy Transfer (FRET) Microscopy

Orai1-GFP (1 μg) was transfected together with 1 μg Orai1-RFP in HEKS1 or alternatively with 2 μg STIM1-mCherry in HEKWT cells and seeded on 25 mm glass coverslips 24 h before measurements. Cells were subjected to Tg-mediated store depletion without (control) or with 10 min pretreatment with 1 mM H_2_O_2_. A Leica AM TIRF MC system was used for FRET measurement within the TIRF focal plane as set to acceptor fluorescence. Three sets of images (GFP, FRET, and RFP) were acquired: GFP was excited using a 488 nm laser (suppression filter BP 525/50); for RFP the laser excitation wavelength was 561 nm (suppression filter BP 600/40), and for FRET image a 488 nm laser was used (suppression filter BP 600/40). Image acquisition and analysis were performed with Leica Application suite, Widefield FRET sensitized emission (SE) module. Acquisition parameters (laser intensity 60%, exposure time 100 ms, penetration depth 150 nm) were held constant for all three channels. The apparent FRET efficiency (E_FRET_) was calculated from background-subtracted images using E_FRET_ = 
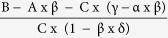
 described by^44^, where A, B and C stand for donor-, FRET- and acceptor channel respectively. Bleed-through and cross talk factors (α, β, γ, δ) were determined individually for every experimental day using cells transfected with either acceptor or donor constructs. To eliminate variation in FRET signal due to expression level, only clusters with acceptor/donor ratio of mean ± standard deviation of the respective experimental day were included in the analysis. (Leica Application suite Software, 2.4.1 build 6384).

### Total Internal Reflection Fluorescence (TIRF) Microscopy

HEKS1 were transfected as above with Orai1-GFP and 24 h later incubated with 0.5 Ca^2+^ Ringer solution with or without 10 min pretreatment with 1 mM H_2_O_2_. On a Leica AM TIRF MC system, cells were mounted for recording fluorescence as in^36^. The stores were depleted by perfusion of 1 μM Tg in 0 Ca^2+^ Ringer solution. Images were analysed with Fiji^45^.

### Statistical analysis

Data obtained are presented as mean ± s.e.m. Statistical significance was tested by performing unpaired, two-tailed Student t-test. Asterisks indicate significant differences. *p < 0.05, **p < 0.01, ***p < 0.001.

### Molecular Modeling and Dynamics

Homology model of human Orai1: We used standard modelling techniques implemented in MOE2015.1001 (Molecular Operating Environment 2008; Chemical Computing Group, Montreal, QC, Canada) to generate a homology model of the human Orai1 channel based on the X-ray structure of Drosophila ORAI1^38^. The Protein Data Bank entry 4HKR, that revealed a hexameric channel, was used as the template. The target protein sequence of hORAI1 was retrieved from the UniProt database with accession number Q96D31. Subsequent sequence alignment between the template and the model sequence was performed using a modified version of the alignment algorithm originally introduced by^46^. In this approach, alignments are computed by optimizing a function based on residue similarity scores. The function uses the amino acid substitution matrix BLOSUM62^47^. The resulting sequence alignment has been manually adjusted according to available site directed mutagenesis data. The presented homology model coordinates are based on the best scoring of 250 generated intermediate models and the overall structural quality confirmed by a Ramachandran plot. The intermediate homology models were refined with the Amber99 force field^48^ using a fine gradient, which was terminated when the root-mean-square was 0.1 Å. The electrostatic solvation energy was used to score the initial 250 generated models. It was calculated using a generalized born/volume integral method^49^. The protonation of the final model was done using the Protonate3D algorithm followed by minimization with a root-mean square of 0.5 Å^50^. This homology model was used for subsequent molecular dynamics simulations using the Nose-Poincare-Andersen equations^51^,^52^ with an equilibrium stage of 100 ps followed by a 50 ns run using a stepsize of 0.5 ps at 300 K. The protein-protein docking module was used within our modeling program (MOE, CCG) with some manual adjustment since the crystallographic information from the hOrai structure has no information on the loops. The human STIM1 SOAR domain (PDB: 3TEQ) coordinates were obtained from^53^. The Amber:EHT force field was implemented in the MOE program to setup the calculation without any further modifications. The input coordinates have been taken from our homology model of hOrai1 that is in the closed state followed by docking of the hSTIM1 SOAR domain. We have simulated without any further modification (full-atom simulation) like forced movements or fields in the sense of a steered MD.

## Additional Information

**How to cite this article**: Alansary, D. *et al*. Thiol dependent intramolecular locking of Orai1 channels. *Sci. Rep*. **6**, 33347; doi: 10.1038/srep33347 (2016).

## Supplementary Material

Supplementary Information
