# Supplementary material for: Thiol dependent intramolecular locking of Orai1 channels
Source: Sci Rep. 2016 Sep 14;6:33347. doi: 10.1038/srep33347 (PMC5022029; doi:10.1038/srep33347)
Supplement: Supplementary Information [file srep33347-s1.pdf]

Thiol dependent intramolecular locking of Orai1 channels

Dalia Alansary[1], Barbara Schmidt[1,2,3], Kathrin Doerr[1], Ivan Bogeski[2], Heiko Rieger[3,] Achim Kless[4], Barbara A. Niemeyer[1]

[1]Molecular Biophysics, Saarland University, 66421 Homburg, Germany

[2] Department of Biophysics, Saarland University, 66421 Homburg, Germany

[3] Department of Theoretical Physics, Saarland University, 66041 Saarbrücken, Germany

[4] Gruenenthal Innovation, Drug Discovery Technologies, Gruenenthal GmbH, 52078 Aachen, Germany

Corresponding author:

Barbara A. Niemeyer, Department of Molecular Biophysics, Center for Integrative Physiology and Molecular Medicine, Building. 48.

Saarland University, 66421 Homburg, Germany.

e-mail: barbara.niemeyer@uks.eu; tel: +49 6841 16 16304; fax: +49 6841 1616302

Running title: Locking Orai1 channels

Keywords: Oxidomimetic, SOCE, reaction-diffusion, molecular dynamics, cysteine

Fig-S1 (Niemeyer)

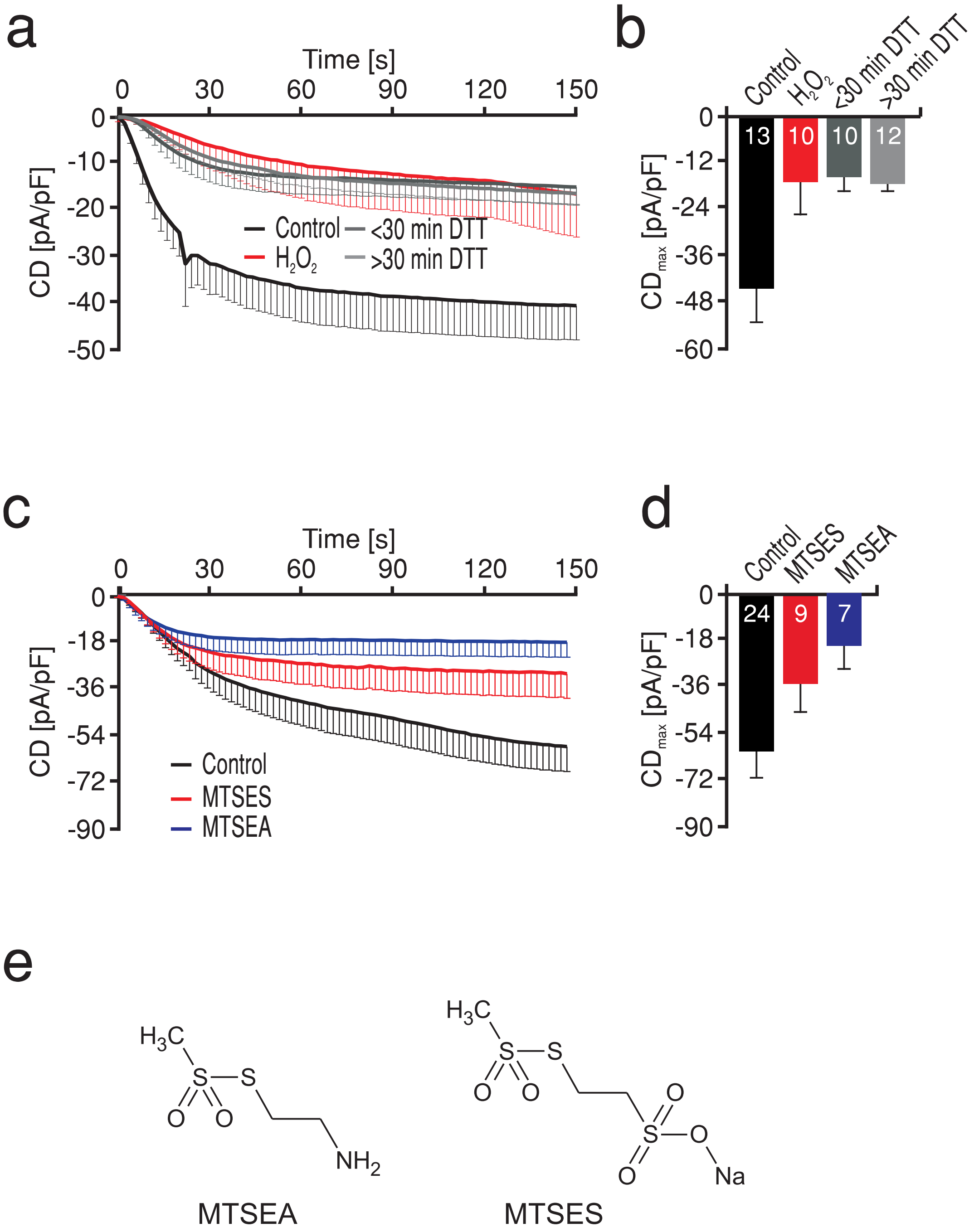

Fig-S2 (Niemeyer)

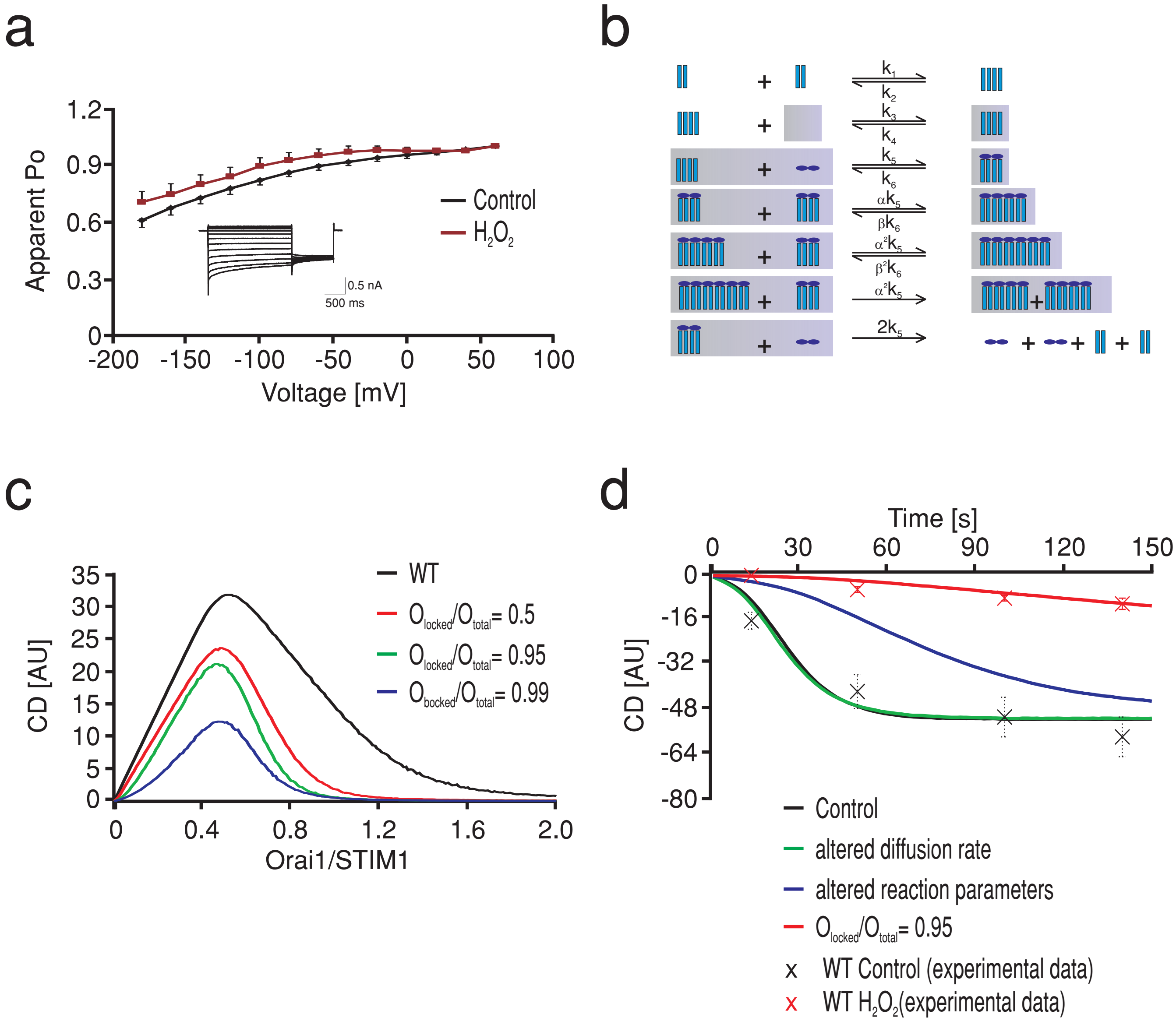

Supplementary Figure Legends

Figure S1. Inhibition of Orai1 mediated I_{CRAC} is irreversible and less accessible to MTSES

(a) Average traces showing whole cell current density (CD) over time extracted at -130mV in HEKS1 cells transfected with 1 µg WT Orai1, measured in control condition (black) or from cells treated only with 1 mM H_2O_2 for 10 min (red) or followed by incubation with DTT for less (dark grey) or more (light grey) than 30 min before current recording. (b) Average CD_{max} recorded from cells measured in a. (c) Average traces showing CD development over time from HEKS1 cells transfected as in a and treated with 500 µM MTSES (red) or 500 µM MTSEA (blue) for 10-15 min before current recording and compared to control treated cells (black). (d) Average CD_{max} recorded from cells measured in c. (e) Chemical structure of MTSEA and MTSES.

Figure S2 Effects of ROS on apparent open probability and reaction diffusion model.

(a) Traces showing apparent open probability (P_o) of Orai1 channels expressed in HEKS1 cells and measured under control conditions (black, n=6) or after treatment with H_2O_2 (red, n=6). The inset shows example traces depicting the resulting tail currents during a step voltage to -100 mV from cells subjected to voltage steps of 20 mV from -160 to +80 mV over 100 ms. (b) Schematic representation of the stepwise multimerization of STIM1 dimers, translocation of multimers to plasma membrane junction regions (grey boxes), recruitment of Orai1 dimers, trapping of STIM1-Orai1 complexes into junctional regions and possible dissociation of STIM1 and Orai1 from focal activation regions. (c) Traces showing change of I_{CRAC} as a function of Orai1 to STIM1 protein ratio as predicted from the stochastic reaction diffusion model under control conditions (WT, black) compared to predicted currents upon heteromeric channel formation between oxidized (locked) and non-oxidized subunits. Coloured traces represent different fractions of inhibited channels: 0.5 (red), 0.95 (green) and 0.99 (blue). (d) Traces showing predicted current development over time in control conditions (black) and under the influence of a slowed diffusion (green, altered diffusion rate), after implementation of all altered reaction parameters in the stochastic reaction diffusion model (blue, altered reaction parameters) and upon inclusion of locked channel subunits at a fraction of 0.95 (red, O_{locked}/O_{total} = 0.95). Representative points of experimentally measured currents in control conditions and after treatment with H_2O_2 are depicted as black and red crosses, respectively.

Supplementary Methods

The stochastic reaction-diffusion model

Similar to [1,2] we simulated the interaction between STIM1 and Orai1 proteins in a stochastic reaction-diffusion model using Gillespie's algorithm [3] with some technical extensions [4]. The model now accounts for multiple ER-plasma membrane junctional regions (grid cells named PMJ) of different sizes into which both, STIM1 oligomers and Orai1 dimers, are able to diffuse, which is described as hopping to a neighbouring grid cell. The hopping constant k_D is derived from the diffusional constant D and the length of a grid cell squared l^2: $k_D = D / l^2$. While interaction of Orai1 and STIM1 is confined to PMJs, oligomerization of STIM1 can take place outside these areas.

In total an area of a (100 × 100) grid cells (sample volumes, SV) is modelled, representing an area of 0.1 µm × 0.1 µm, and continuous boundary conditions were adopted. Within the PMJ the following reactions are allowed: STIM1 dimers are able to form oligomers in form of dimers of dimers, these STIM1 oligomers are able to anchor to the PMJ and become stationary (k_D = 0 1/s) where they trap Orai1 dimers. The model assumes non-cooperative interaction of Orai1 subunits accounted for by introducing factors α and β to the reaction constants (Table S2). One CRAC channel complex consists of one STIM1 dimer of dimers and one Orai1 dimer and cannot diffuse out of its subvolume. Two of these channel complexes form an intermediate CRAC channel state and finally, three subunits build up a fully open hexameric channel. Figure S2b depicts the reaction scheme. The model also introduces a "stealing mechanism" to represent a continuous assembly-disassembly of the channel states (reactions in lines 6 and 7 of Fig S2b). I_{CRAC} is predicted by forming a linear combination of all CRAC channel states weighted with different open probabilities.

For different ratios of Orai1/STIM1 the model predicts an altered current density at steady state (600 s) with a maximum at a ratio of 0.5 (Fig S2c, see also [1]).

In addition, the model was modified to introduce and account for the locked channel subunits (O_H) that can replace one or more open subunit in the channel complex. The different combinations of locked to open channel subunits result in species with graded states of conductivity. The model predicts the current density from the sum of the number of sub-states (N) multiplied by the corresponding conductivity (c) and weighted by a new constant δ that accounts for current inhibition (see equations below). The steady-state values of I_{CRAC} resulting by changing the ratio of locked to total Orai1 O_H / O_{tot} show the same tendency to be affected by Orai1/STIM1 ratio whereby the current density is smaller with higher fraction of locked Orai1 (Fig S2c).

$$
\begin{aligned}
I_{CRAC} = {} & c_1 N_{|O\rangle} + c_2 N_{|OO\rangle} + c_3 N_{|OOO\rangle} \\
& + \delta\left(c_1 N_{|\,O_H\,\rangle} + c_2 N_{|O_H O_H} + c_3 N_{|O_H O_H O_H\rangle}\right) \\
& + \frac{c_2}{2}(1+\delta) N_{|OO_H\rangle} \\
& + \frac{c_3}{3}(2+\delta) N_{|OOO_H\rangle} + \frac{c_3}{3}(1+2\delta) N_{|OO_H O_H\rangle}
\end{aligned}
$$

With $c_1 = 0.125$, $c_2 = 0.5$ and $c_3 = 1$

**Species**	**Abbreviation**	**Diffusion constant in µm^2/s**	**Hopping rate in 1/s**
Proteins			
STIM dimer	S_2	0.1	10
Dimer of STIM dimers	S_4	0.05	5
Dimer of STIM dimers attached to the PM	K_4	0	0
Orai dimer	O_2	0.07	7
H_2O_2 inhibited Orai dimer	$O_{2,H}$	0.07/1.5	7/1.5
Channel subunit composed of			
$O_2 + K_4$	$\|O\rangle$	0	0
$O_{2,H} + K_4$	$\|O_H\ \rangle$	0	0
Intermediate channel state composed of			
$\|O\rangle + \|O\rangle$	$\|OO\rangle$	0	0
$\|O\rangle + \|O_H\rangle$	$\|OO_H\rangle$	0	0
$\|O_H\rangle + \|O_H\rangle$	$\|O_H O_H\rangle$	0	0
Fully open channel composed of			
$\|O\rangle + \|O\rangle + \|O\rangle$	$\|OOO\rangle$	0	0
$\|O\rangle + \|O\rangle + \|O_H\rangle$	$\|OOO_H\rangle$	0	0
$\|O\rangle + \|O_H\rangle + \|O_H\rangle$	$\|OO_H O_H\rangle$	0	0
$\|O_H\rangle + \|O_H\rangle + \|O_H\rangle$	$\|O_H O_H O_H\rangle$	0	0

Table S1

List of all SOCE components introduced in the stochastic model including their diffusion constant and the corresponding hopping rate.

Reaction	Rate
$S_2 + S_2 \rightarrow S_4$	k_1
$S_4 \rightarrow S_2 + S_2$	k_2
$S_4 + PMJ \rightarrow K_4 + PMJ$	k_3
$K_4 + PMJ \rightarrow S_4 + PMJ$	k_4
$K_4 + O_2 \rightarrow \vert O\rangle$	k_5
$\vert O\rangle \rightarrow K_4 + O_2$	k_6
$\vert O\rangle + \vert O\rangle \rightarrow \vert OO\rangle$	$k_7 = \alpha k_5$
$\vert OO\rangle \rightarrow \vert O\rangle + \vert O\rangle$	$k_8 = \beta k_6$
$\vert OO\rangle + \vert O\rangle \rightarrow \vert OOO\rangle$	$k_9 = \alpha^2 k_5$
$\vert OOO\rangle \rightarrow \vert OO\rangle + \vert O\rangle$	$k_{10} = \beta^2 k_6$
$K_4 + O_{2,H} \rightarrow \vert O_H\rangle$	$\widetilde{k_5} = k_5 \cdot 1{,}4$
$\vert O_H\rangle \rightarrow K_4 + O_{2,H}$	$\widetilde{k_6} = k_6 \cdot 1{,}4$
$\vert O_H\rangle + \vert O_H\rangle \rightarrow \vert O_H\rangle$	$\widetilde{k_7} = k_7 \cdot 1{,}4/2.25$
$\vert O_H O_H\rangle \rightarrow \vert O_H\rangle + \vert O_H\rangle$	$\widetilde{k_8} = k_8 \cdot 1{,}4/2.25$
$\vert O_H O_H\rangle + \vert O_H\rangle \rightarrow \vert O_H O_H O_H\rangle$	$\widetilde{k_9} = k_9 \cdot 1{,}4/2.25$
$\vert O_H O_H O_H\rangle \rightarrow \vert O_H O_H\rangle + \vert O_H\rangle$	$\widetilde{k_{10}} = k_{10} \cdot 1{,}4/2.25$
$\vert O\rangle + \vert O_H\rangle \rightarrow \vert OO_H\rangle$	$\widetilde{k_7}$
$\vert OO_H\rangle \rightarrow \vert O\rangle + \vert O_H\rangle$	$\widetilde{k_8}$
$\vert OO\rangle + \vert O_H\rangle \rightarrow \vert OOO_H\rangle$	$\widetilde{k_9}$
$\vert OOO_H\rangle \rightarrow \vert OO\rangle + \vert O_H\rangle$	$\widetilde{k_{10}}$
$\vert OO_H\rangle + \vert O_H\rangle \rightarrow \vert OO_H O_H\rangle$	$\widetilde{k_9}$
$\vert OO_H O_H\rangle \rightarrow \vert OO_H\rangle + \vert O_H\rangle$	$\widetilde{k_{10}}$
$\vert O_H O_H\rangle + \vert O\rangle \rightarrow \vert OO_H O_H\rangle$	$\widetilde{k_9}$
$\vert OO_H O_H\rangle \rightarrow \vert O_H O_H\rangle + \vert O\rangle$	$\widetilde{k_{10}}$
$\vert OOO\rangle + \vert O\rangle \rightarrow \vert OO\rangle + \vert OO\rangle$	k_9
$\vert O\rangle + O_2 \rightarrow O_2 + O_2 + S_2 + S_2$	$k_{11} = 2k_5$
$\vert O_H O_H O_H\rangle + \vert O_H\rangle \rightarrow \vert O_H O_H\rangle + \vert O_H O_H\rangle$	$\widetilde{k_9}$
$\vert O_H\rangle + O_{2,H} \rightarrow O_{2,H} + O_{2,H} + S_2 + S_2$	$\widetilde{k_{11}} = k_{11} \cdot 1{,}4$
$\vert OOO\rangle + \vert O_H\rangle \rightarrow \vert OO\rangle + \vert OO_H\rangle$	$\widetilde{k_9}$
$\vert OOO_H\rangle + \vert O\rangle \rightarrow \vert OO\rangle + \vert OO_H\rangle$	$\widetilde{k_9}$
$\vert OOO_H\rangle + \vert O_H\rangle \rightarrow \vert OO_H\rangle + \vert OO_H\rangle$	$\widetilde{k_9}$
$\vert OOO_H\rangle + \vert O_H\rangle \rightarrow \vert OO\rangle + \vert O_H O_H\rangle$	$\widetilde{k_9}$
$\vert OO_H O_H\rangle + \vert O\rangle \rightarrow \vert OO_H\rangle + \vert OO_H\rangle$	$\widetilde{k_9}$
$\vert OO_H O_H\rangle + \vert O\rangle \rightarrow \vert OO\rangle + \vert O_H O_H\rangle$	$\widetilde{k_9}$
$\vert OO_H O_H\rangle + \vert O_H\rangle \rightarrow \vert OO_H\rangle + \vert O_H O_H\rangle$	$\widetilde{k_9}$
$\vert O_H O_H O_H\rangle + \vert O\rangle \rightarrow \vert OO_H\rangle + \vert O_H O_H\rangle$	$\widetilde{k_9}$
$\vert O\rangle + O_{2,H} \rightarrow O_2 + O_{2,H} + S_2 + S_2$	$\widetilde{k_{11}}$
$\vert O_H\rangle + O_2 \rightarrow OO_{2,H} + O_2 + S_2 + S_2$	$\widetilde{k_{11}}$

Table S2

A full list of all possible channel configurations and corresponding rates

Parameter	Value
k_1	$4.8 \cdot 10^5 \frac{m^3}{mol \cdot s}$
k_2	$0.01 \frac{1}{s}$
k_3	$1.8 \cdot 10^6 \frac{m^3}{mol \cdot s}$
k_4	$0.3 \frac{1}{s}$
k_5	$4.8 \cdot 10^5 \frac{m^3}{mol \cdot s}$
k_6	$0.1 \frac{1}{s}$
α	0.8
β	0.8

Table S3

Corresponding values for the rate constants and the factors α and β

References

1. Kilch, T. *et al.* Mutations of the Ca2+-sensing Stromal Interaction Molecule STIM1 Regulate Ca2+ Influx by Altered Oligomerization of STIM1 and by Destabilization of the Ca2+ Channel Orai1. *J Biol Chem* **288**, 1653-1664, doi:10.1074/jbc.M112.417246 (2013).
2. Peglow, M. N., B.A.; Hoth, H.; Rieger, H. Interplay of channels, pumps and organelle location in calcium microdomain formation. *New Journal of Physics* **15**, doi:10.1088/1367-2630/1015/1085/055022 (2013).
3. Gillespie, D. T. A general method for numerically simulating the stochastic time evolution of coupled chemical reactions. *Journal of Computational Physics* **22**, 403-434 (1976).
4. Gibson, M. A. B., J. Efficient Exact Stochastic Simulation of Chemical Systems with Many Species and Many Channels. *Journal of Physical Chemistry* **A 104** 1876-1889 (2000).
